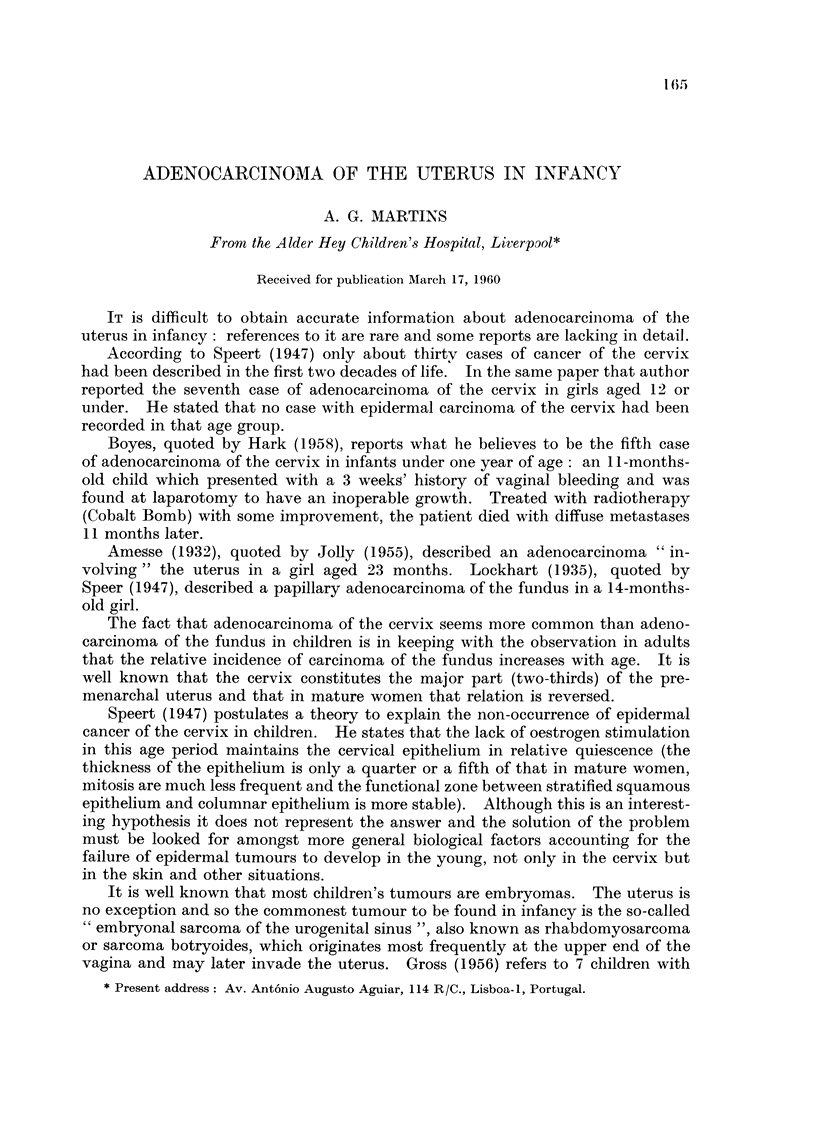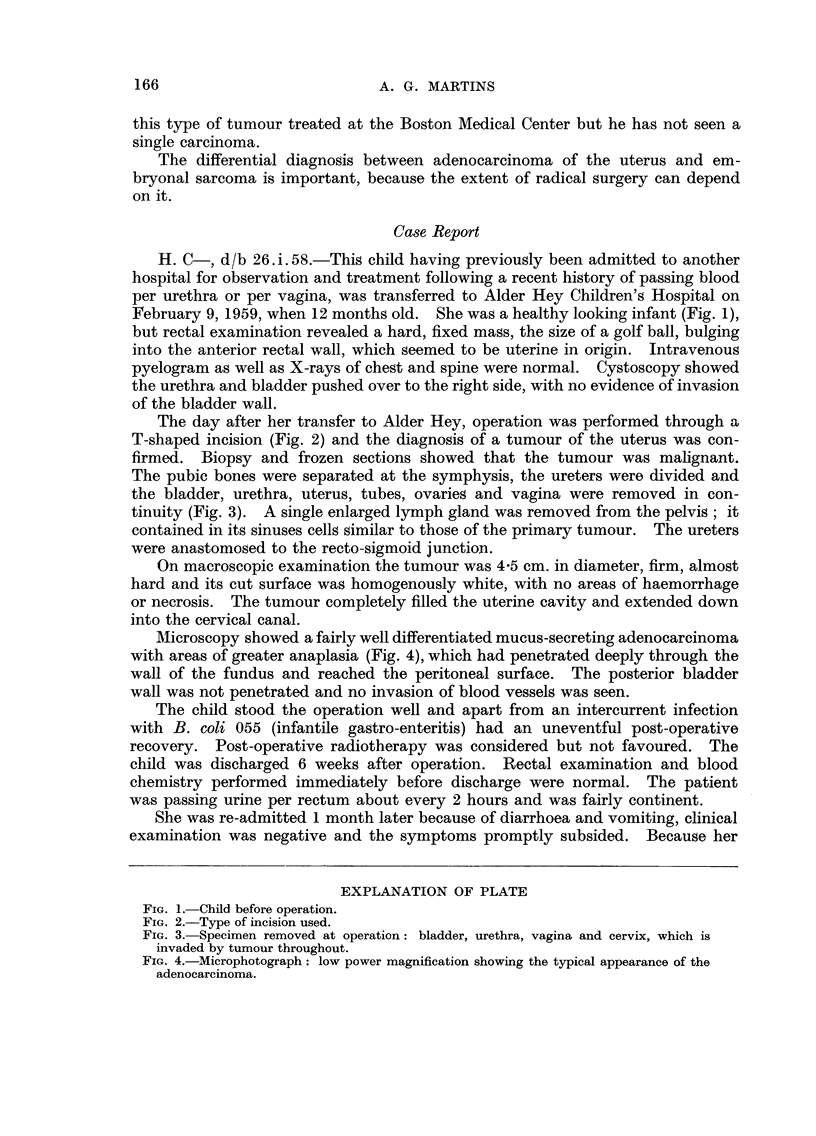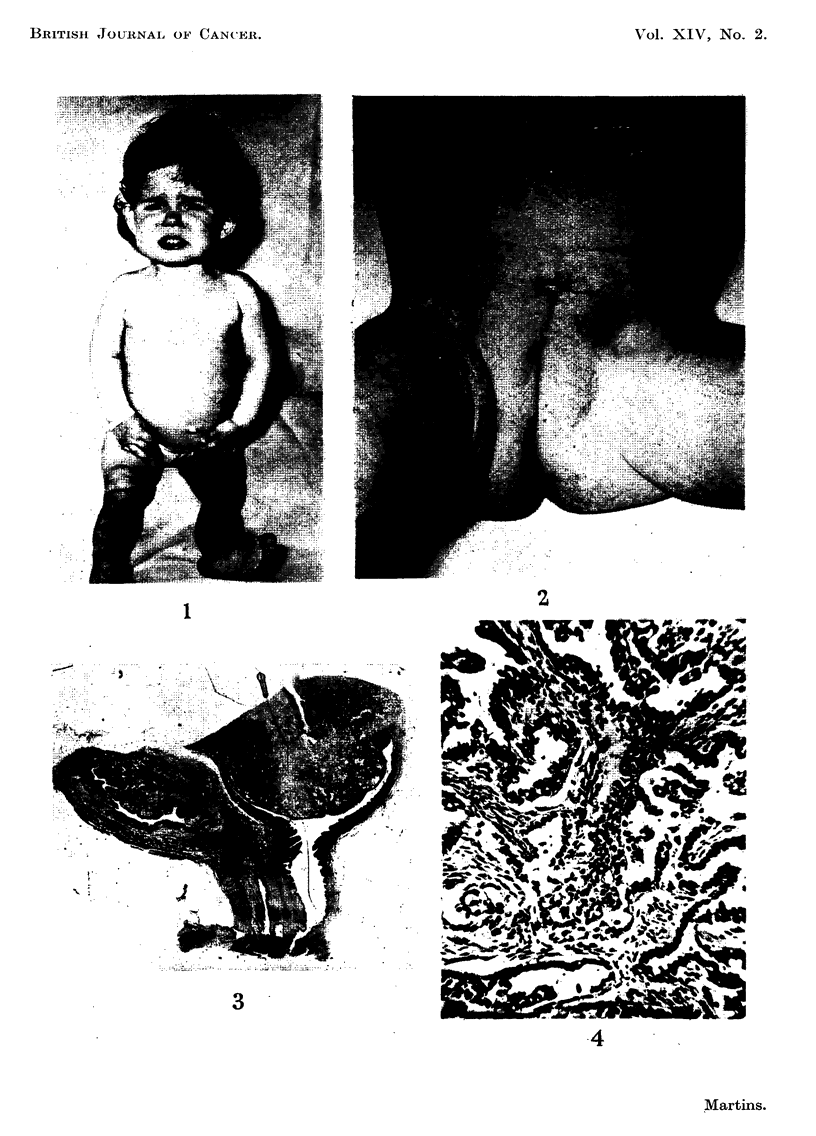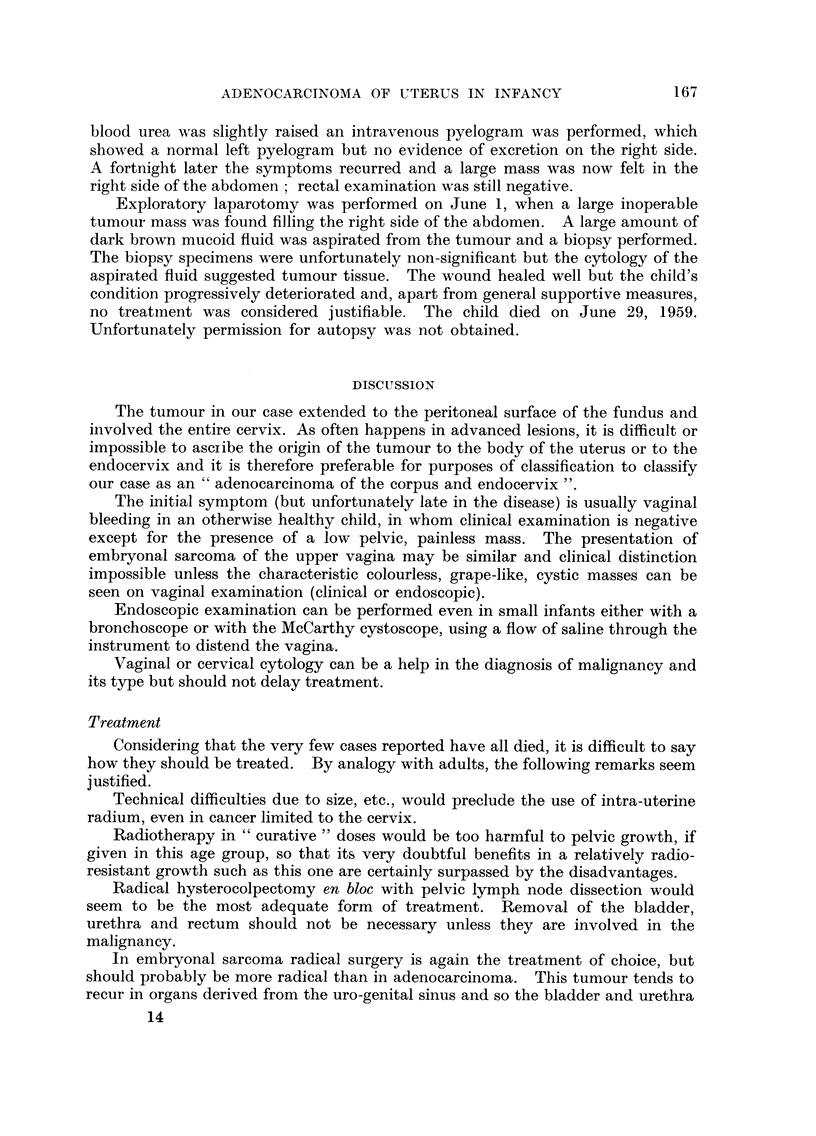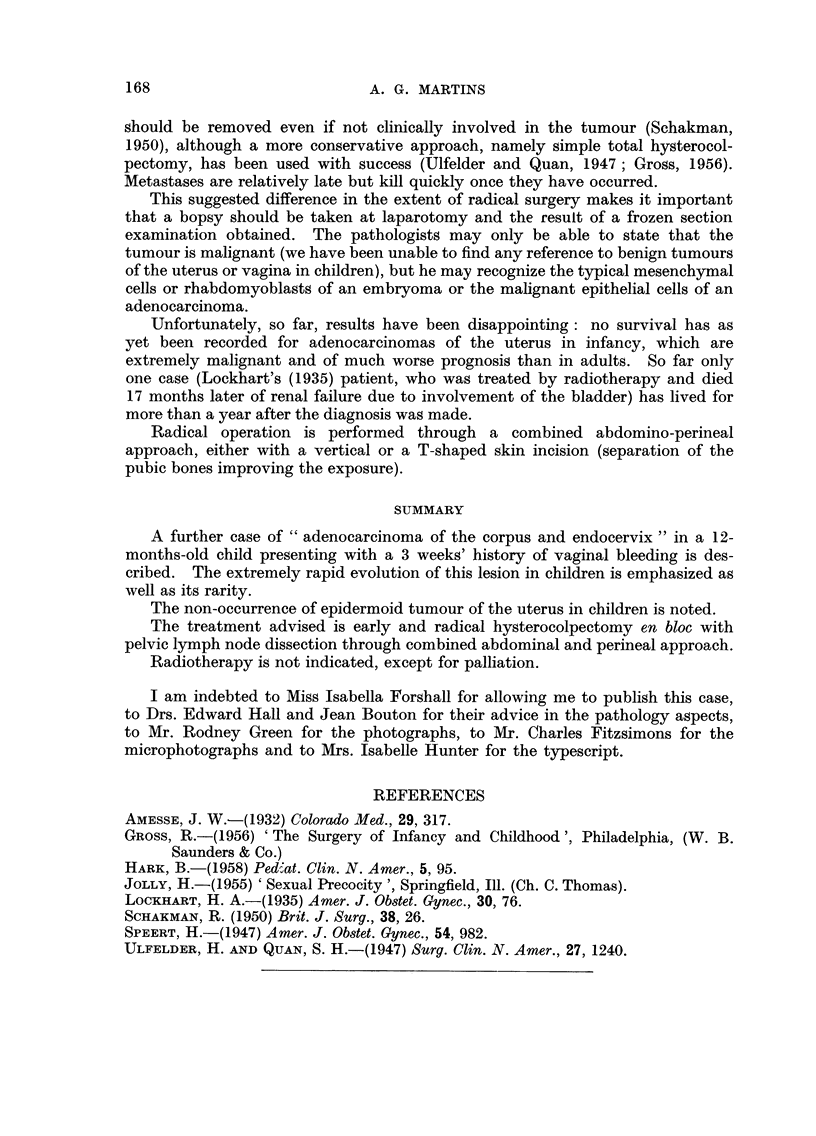# Adenocarcinoma of the Uterus in Infancy

**DOI:** 10.1038/bjc.1960.19

**Published:** 1960-06

**Authors:** A. G. Martins

## Abstract

**Images:**


### ADENOCARCINOMA OF THE UTERUS IN INFANCY

A. G. MARTINS

*From the Alder Hey Children's Hospital, Liverpool**

Received for publication March 17, 1960

IT is difficult to obtain accurate information about adenocarcinoma of the uterus in infancy : references to it are rare and some reports are lacking in detail.

According to Speert (1947) only about thirty cases of cancer of the cervix had been described in the first two decades of life. In the same paper that author reported the seventh case of adenocarcinoma of the cervix in girls aged 12 or under. He stated that no case with epidermal carcinoma of the cervix had been recorded in that age group.

Boyes, quoted by Hark (1958), reports what he believes to be the fifth case of adenocarcinoma of the cervix in infants under one year of age : an 11-months-old child which presented with a 3 weeks' history of vaginal bleeding and was found at laparotomy to have an inoperable growth. Treated with radiotherapy (Cobalt Bomb) with some improvement, the patient died with diffuse metastases 11 months later.

Amesse (1932), quoted by Jolly (1955), described an adenocarcinoma " involving " the uterus in a girl aged 23 months. Lockhart (1935), quoted by Speer (1947), described a papillary adenocarcinoma of the fundus in a 14-months-old girl.

The fact that adenocarcinoma of the cervix seems more common than adenocarcinoma of the fundus in children is in keeping with the observation in adults that the relative incidence of carcinoma of the fundus increases with age. It is well known that the cervix constitutes the major part (two-thirds) of the pre-menarchal uterus and that in mature women that relation is reversed.

Speert (1947) postulates a theory to explain the non-occurrence of epidermal cancer of the cervix in children. He states that the lack of oestrogen stimulation in this age period maintains the cervical epithelium in relative quiescence (the thickness of the epithelium is only a quarter or a fifth of that in mature women, mitosis are much less frequent and the functional zone between stratified squamous epithelium and columnar epithelium is more stable). Although this is an interesting hypothesis it does not represent the answer and the solution of the problem must be looked for amongst more general biological factors accounting for the failure of epidermal tumours to develop in the young, not only in the cervix but in the skin and other situations.

It is well known that most children's tumours are embryomas. The uterus is no exception and so the commonest tumour to be found in infancy is the so-called " embryonal sarcoma of the urogenital sinus ", also known as rhabdomyosarcoma or sarcoma botryoides, which originates most frequently at the upper end of the vagina and may later invade the uterus. Gross (1956) refers to 7 children with

* Present address : Av. António Augusto Aguiar, 114 R/C., Lisboa-1, Portugal.

this type of tumour treated at the Boston Medical Center but he has not seen a single carcinoma.

The differential diagnosis between adenocarcinoma of the uterus and embryonal sarcoma is important, because the extent of radical surgery can depend on it.

### *Case Report*

H. C—, d/b 26.i.58.—This child having previously been admitted to another hospital for observation and treatment following a recent history of passing blood per urethra or per vagina, was transferred to Alder Hey Children's Hospital on February 9, 1959, when 12 months old. She was a healthy looking infant (Fig. 1), but rectal examination revealed a hard, fixed mass, the size of a golf ball, bulging into the anterior rectal wall, which seemed to be uterine in origin. Intravenous pyelogram as well as X-rays of chest and spine were normal. Cystoscopy showed the urethra and bladder pushed over to the right side, with no evidence of invasion of the bladder wall.

The day after her transfer to Alder Hey, operation was performed through a T-shaped incision (Fig. 2) and the diagnosis of a tumour of the uterus was confirmed. Biopsy and frozen sections showed that the tumour was malignant. The pubic bones were separated at the symphysis, the ureters were divided and the bladder, urethra, uterus, tubes, ovaries and vagina were removed in continuity (Fig. 3). A single enlarged lymph gland was removed from the pelvis ; it contained in its sinuses cells similar to those of the primary tumour. The ureters were anastomosed to the recto-sigmoid junction.

On macroscopic examination the tumour was 4·5 cm. in diameter, firm, almost hard and its cut surface was homogenously white, with no areas of haemorrhage or necrosis. The tumour completely filled the uterine cavity and extended down into the cervical canal.

Microscopy showed a fairly well differentiated mucus-secreting adenocarcinoma with areas of greater anaplasia (Fig. 4), which had penetrated deeply through the wall of the fundus and reached the peritoneal surface. The posterior bladder wall was not penetrated and no invasion of blood vessels was seen.

The child stood the operation well and apart from an intercurrent infection with *B. coli* 055 (infantile gastro-enteritis) had an uneventful post-operative recovery. Post-operative radiotherapy was considered but not favoured. The child was discharged 6 weeks after operation. Rectal examination and blood chemistry performed immediately before discharge were normal. The patient was passing urine per rectum about every 2 hours and was fairly continent.

She was re-admitted 1 month later because of diarrhoea and vomiting, clinical examination was negative and the symptoms promptly subsided. Because her

---

EXPLANATION OF PLATE

FIG. 1.—Child before operation.

FIG. 2.—Type of incision used.

FIG. 3.—Specimen removed at operation : bladder, urethra, vagina and cervix, which is invaded by tumour throughout.

FIG. 4.—Microphotograph : low power magnification showing the typical appearance of the adenocarcinoma.

1

2

3

4

Martins.

blood urea was slightly raised an intravenous pyelogram was performed, which showed a normal left pyelogram but no evidence of excretion on the right side. A fortnight later the symptoms recurred and a large mass was now felt in the right side of the abdomen ; rectal examination was still negative.

Exploratory laparotomy was performed on June 1, when a large inoperable tumour mass was found filling the right side of the abdomen. A large amount of dark brown mucoid fluid was aspirated from the tumour and a biopsy performed. The biopsy specimens were unfortunately non-significant but the cytology of the aspirated fluid suggested tumour tissue. The wound healed well but the child's condition progressively deteriorated and, apart from general supportive measures, no treatment was considered justifiable. The child died on June 29, 1959. Unfortunately permission for autopsy was not obtained.

### DISCUSSION

The tumour in our case extended to the peritoneal surface of the fundus and involved the entire cervix. As often happens in advanced lesions, it is difficult or impossible to ascribe the origin of the tumour to the body of the uterus or to the endocervix and it is therefore preferable for purposes of classification to classify our case as an " adenocarcinoma of the corpus and endocervix ".

The initial symptom (but unfortunately late in the disease) is usually vaginal bleeding in an otherwise healthy child, in whom clinical examination is negative except for the presence of a low pelvic, painless mass. The presentation of embryonal sarcoma of the upper vagina may be similar and clinical distinction impossible unless the characteristic colourless, grape-like, cystic masses can be seen on vaginal examination (clinical or endoscopic).

Endoscopic examination can be performed even in small infants either with a bronchoscope or with the McCarthy cystoscope, using a flow of saline through the instrument to distend the vagina.

Vaginal or cervical cytology can be a help in the diagnosis of malignancy and its type but should not delay treatment.

### *Treatment*

Considering that the very few cases reported have all died, it is difficult to say how they should be treated. By analogy with adults, the following remarks seem justified.

Technical difficulties due to size, etc., would preclude the use of intra-uterine radium, even in cancer limited to the cervix.

Radiotherapy in " curative " doses would be too harmful to pelvic growth, if given in this age group, so that its very doubtful benefits in a relatively radio-resistant growth such as this one are certainly surpassed by the disadvantages.

Radical hysterocolpectomy *en bloc* with pelvic lymph node dissection would seem to be the most adequate form of treatment. Removal of the bladder, urethra and rectum should not be necessary unless they are involved in the malignancy.

In embryonal sarcoma radical surgery is again the treatment of choice, but should probably be more radical than in adenocarcinoma. This tumour tends to recur in organs derived from the uro-genital sinus and so the bladder and urethra should be removed even if not clinically involved in the tumour (Schakman, 1950), although a more conservative approach, namely simple total hysterocolpectomy, has been used with success (Ulfelder and Quan, 1947 ; Gross, 1956). Metastases are relatively late but kill quickly once they have occurred.

This suggested difference in the extent of radical surgery makes it important that a bopsy should be taken at laparotomy and the result of a frozen section examination obtained. The pathologists may only be able to state that the tumour is malignant (we have been unable to find any reference to benign tumours of the uterus or vagina in children), but he may recognize the typical mesenchymal cells or rhabdomyoblasts of an embryoma or the malignant epithelial cells of an adenocarcinoma.

Unfortunately, so far, results have been disappointing : no survival has as yet been recorded for adenocarcinomas of the uterus in infancy, which are extremely malignant and of much worse prognosis than in adults. So far only one case (Lockhart's (1935) patient, who was treated by radiotherapy and died 17 months later of renal failure due to involvement of the bladder) has lived for more than a year after the diagnosis was made.

Radical operation is performed through a combined abdomino-perineal approach, either with a vertical or a T-shaped skin incision (separation of the pubic bones improving the exposure).

### SUMMARY

A further case of " adenocarcinoma of the corpus and endocervix " in a 12-months-old child presenting with a 3 weeks' history of vaginal bleeding is described. The extremely rapid evolution of this lesion in children is emphasized as well as its rarity.

The non-occurrence of epidermoid tumour of the uterus in children is noted.

The treatment advised is early and radical hysterocolpectomy *en bloc* with pelvic lymph node dissection through combined abdominal and perineal approach.

Radiotherapy is not indicated, except for palliation.

I am indebted to Miss Isabella Forshall for allowing me to publish this case, to Drs. Edward Hall and Jean Bouton for their advice in the pathology aspects, to Mr. Rodney Green for the photographs, to Mr. Charles Fitzsimons for the microphotographs and to Mrs. Isabelle Hunter for the typescript.

### REFERENCES

AMESSE, J. W.—(1932) *Colorado Med.*, **29**, 317.
GROSS, R.—(1956) 'The Surgery of Infancy and Childhood', Philadelphia, (W. B. Saunders & Co.)
HARK, B.—(1958) *Pediat. Clin. N. Amer.*, **5**, 95.
JOLLY, H.—(1955) 'Sexual Precocity', Springfield, Ill. (Ch. C. Thomas).
LOCKHART, H. A.—(1935) *Amer. J. Obstet. Gynec.*, **30**, 76.
SCHAKMAN, R. (1950) *Brit. J. Surg.*, **38**, 26.
SPEERT, H.—(1947) *Amer. J. Obstet. Gynec.*, **54**, 982.
ULFELDER, H. AND QUAN, S. H.—(1947) *Surg. Clin. N. Amer.*, **27**, 1240.